# 

**Published:** 1907-07-15

**Authors:** 


					﻿Gt. ,‘*A; ¿	İ
Dental
Register
NELVILLE S. HOFF, Editor,
Ann Arbor, Mich.
Vol. LXI	—JULY, 1907—	No. 7
SAMUEL A. CROCKER & CO., Publishers
OHIO DENTAL AND SURGICAL DEPOT
35 W. FIFTH STREET, CINCINNATI, 0.
PRICE, ORE DOLLAR A YEAR
Entered at the Postoffice at Cincinnati O., as second-class mail matter.
				

